# A key role for TGF-β1 in hippocampal synaptic plasticity and memory

**DOI:** 10.1038/srep11252

**Published:** 2015-06-10

**Authors:** Filippo Caraci, Walter Gulisano, Chiara A. Guida, Agata A. R. Impellizzeri, Filippo Drago, Daniela Puzzo, Agostino Palmeri

**Affiliations:** 1 Department of Drug Sciences, University of Catania, Catania, Italy; 2 IRCCS Associazione Oasi Maria S.S., Institute for Research on Mental Retardation and Brain Aging, Troina, Italy; 3 Department of Biomedical and Biotechnological Sciences - Section of Physiology, University of Catania, Catania, Italy; 4 Section of Pharmacology, University of Catania, Catania, Italy

## Abstract

Transforming Growth Factor β1 (TGF-β1) is a well-known neuroprotective and neurotrophic factor demonstrated to play a role in synaptic transmission. However, its involvement in physiological mechanisms underlying synaptic plasticity and memory at hippocampal level has not been thoroughly investigated. Here, we examine the role of TGF-β1 in hippocampal long-term potentiation (LTP) and memory in adult wild type mice. Our data provide evidence that administration of exogenous TGF-β1 is able to convert early-phase-LTP into late-phase-LTP. Furthermore, we show that the block of the endogenous TGF-β1 signaling pathway by the specific TGF-β1 inhibitor SB431542, impairs LTP and object recognition memory. The latter impairment was rescued by administration of exogenous TGF-β1, suggesting that endogenously produced TGF-β1 plays a role in physiological mechanisms underlying LTP and memory. Finally, TGF-β1 functional effect correlates with an increased expression of the phosphorylated transcription factor cAMP-Responsive Element Binding protein.

Long-term potentiation (LTP), a form of synaptic plasticity consisting in the long-lasting enhancement in efficacy of the synapse, has been identified as the molecular substrate of learning and memory^1^. LTP, like memory, exhibits a protein-synthesis independent early phase (E-LTP), and a protein-synthesis dependent late-phase (L-LTP) underpinned by different mechanisms^2^,^3^. It is well known that cAMP-Responsive-Element-Binding (CREB) is involved in various forms of learning and memory in species ranging from invertebrates to mammals and its phosphorylation at Ser133 is responsible for the increase in memory-associated gene transcription required for L-LTP and long-term memory^4^,^5^,^6^. Several pathways lead to CREB phosphorylation and, among these, here we have focused on the Transforming Growth Factor β1 (TGF-β1) which has been suggested to represent a possible link between activity-dependent and neurotrophin-dependent regulation of neuronal function^7^.

TGF-β1 is a member of TGF-β superfamily, which includes several groups of highly conserved multifunctional cell-cell signaling proteins of key importance in the control of cell growth, differentiation as well as immune suppression and repair after injury^8^. In addition to its neurotrophic and neuroprotective role, TGF-β1 has been recently demonstrated to participate in mechanisms of synaptic transmission and its release and expression from mouse hippocampal neurons is regulated in an activity-dependent fashion^9^,^10^.

In the sea-snail Aplysia Californica, treatment with TGF-β1 enhances long-term facilitation at sensorimotor synapses^11^,^12^ and reduces synaptic depression through activation of MAPK and CREB phosphorylation^7^,^13^. The increase of phospho-CREB (p-CREB) levels was also shown after treatment of mouse hippocampal neurons with TGF-β2, which was able to enhance postsynaptic currents and to increase the amplitude and frequency of miniature potentials^14^.

Based on these findings, here we investigate the role of TGF-β1 in hippocampal synaptic plasticity and memory in wild type mice. For this purpose, we first examine the effect of exogenous TGF-β1 on CA1 LTP and CREB phosphorylation. Then, we investigate the role of endogenous TGF-β1 in hippocampal synaptic plasticity, recognition memory and p-CREB levels by blocking its signaling pathway with SB431542, a selective inhibitor of the activin-like kinase (ALK) TGF-β1 receptors^15^.

## Results

### TGF-β1 converts E-LTP in L-LTP in hippocampal CA1 area

**Figure 1:**
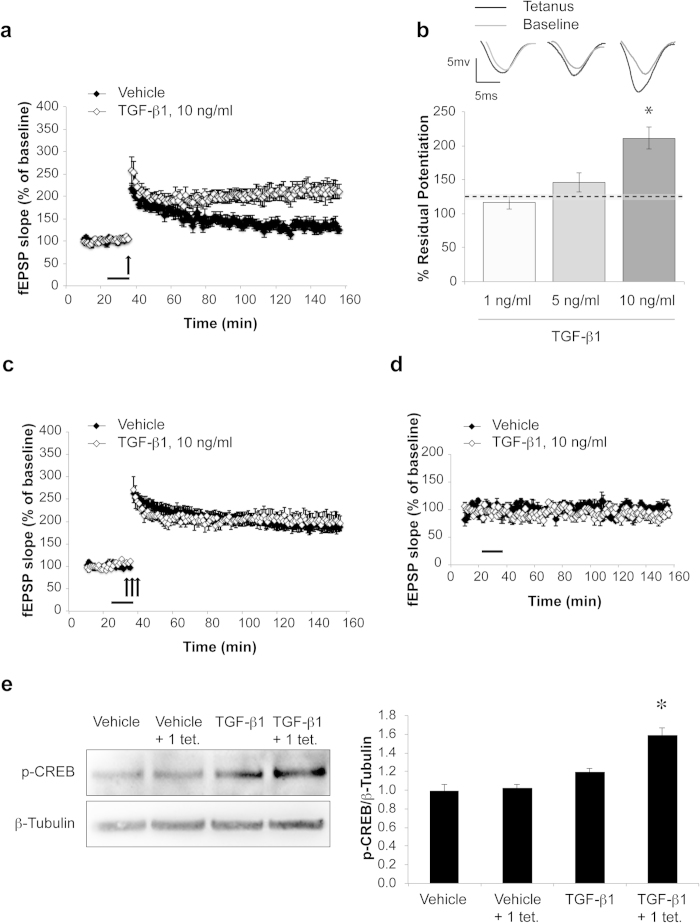
TGF-β1 induces late-LTP and increases CREB phosphorylation. (**a**) A perfusion with TGF-β1 (10 ng/ml) before a weak tetanus converts E-LTP into L-LTP. Bars represent the time of application of drugs and arrows indicate tetanus delivery in this and following figures (1 arrow = weak tetanus; 3 arrows = strong tetanus). (**b**) Lower concentrations of TGF-β1 do not affect potentiation. In the upper part, traces of LTP before (grey line) and after tetanus (black line). (**c**) TGF-β1 (10 ng/ml) does not affect L-LTP induced by a strong tetanus or (**d**) baseline transmission. (**e**) Western blot analyses shows that a treatment with TGF-β1 for 10 min before a weak tetanus significantly increased CREB phosphorylation. Slices treated with TGF-β1 alone show a slight increase of p-CREB, whereas no p-CREB changes are observed in vehicle-treated tetanized slices after a weak-tetanus or in slices treated with vehicle alone. β-tubulin expression is shown as an internal control. * significant difference (p < 0.05).

To evaluate whether TGF-β1 might affect synaptic plasticity we performed electrophysiological experiments on hippocampal slices *in vitro*. We stimulated Schaffer collateral fibers by using a weak tetanus (1 tetanus consisting of a 10-burst train of 4 pulses at 100 Hz with the bursts repeated at 5 Hz) to produce E-LTP in CA1 stratum radiatum. Ten minutes perfusion of hippocampal slices with TGF-β1 at a concentration of 10 ng/ml^7^,^16^ before a weak tetanus produced a late-LTP (n = 7/7; 211.06 ± 15.92% vs. 125.08 ± 8.74% of baseline slope 120 min after tetanus in vehicle-treated slices; ANOVA for repeated measures of the post-tetanic time points: F_(1,12)_ = 11.02, p = 0.006; Fig. 1a), whereas no effects were observed with lower concentrations of TGF-β1 (n = 7 for each condition; 5 ng/ml: 146.15 ± 14.30% of baseline slope 120 min after tetanus; 1 ng/ml: 115.84 ± 9.19% of baseline slope 120 min after tetanus; ANOVA comparing the averages of the 120° min of LTP recording in slices treated with different doses of TGF-β1 and vehicle: F_(3, 24)_ = 11.907, p < 0.0001; Bonferroni post hoc test: p < 0.0001 comparing TGF-β1 10 ng/ml with vehicle or other concentrations used; Fig. 1b).

We then used a strong theta-burst tetanus (3 tetani consisting of a 10-burst train of 4 pulses at 100 Hz with the bursts repeated at 5 Hz) to induce L-LTP. Perfusion with TGF-β1 (10 ng/ml) before a strong tetanus did not affect potentiation (n = 6/6; 197.02 ± 11.34 vs. 187.52 ± 20.60% of baseline slope 120 min after tetanus in vehicle-treated tetanized slices; ANOVA for repeated measures of the post-tetanic time points: F_(1,10)_ = 0.002, p = 0.962; Fig. 1c). Moreover, TGF-β1 did not modify baseline transmission (n = 6/6; 92.02 ± 8.22 vs. 94.85 ± 6.94% of baseline slope; ANOVA for repeated measures: F_(1,10)_ = 1.88, p = 0.200; Fig. 1d).

To investigate the molecular correlate of the TGF-β1-induced increase in LTP, we turned to the transcription factor CREB, involved in various forms of synaptic plasticity and memory^4^,^5^,^6^. We used western blot to analyze phospho-CREB (p-CREB) levels on the same hippocampal slices used for electrophysiological experiments. In slices treated with TGF-β1 for 10 min before a weak tetanus, a 56% increase in CREB phosphorylation was found (1.58 ± 0.07 vs. 1.01 ± 0.04; Bonferroni post hoc: p = 0.001; two-way ANOVA for the interaction between tetanization and TGF-β1 treatment: F_(2, 6)_ = 25.470, p = 0.001; Fig. 1e).

### Endogenous TGF-β1 plays a role in hippocampal LTP

**Figure 2:**
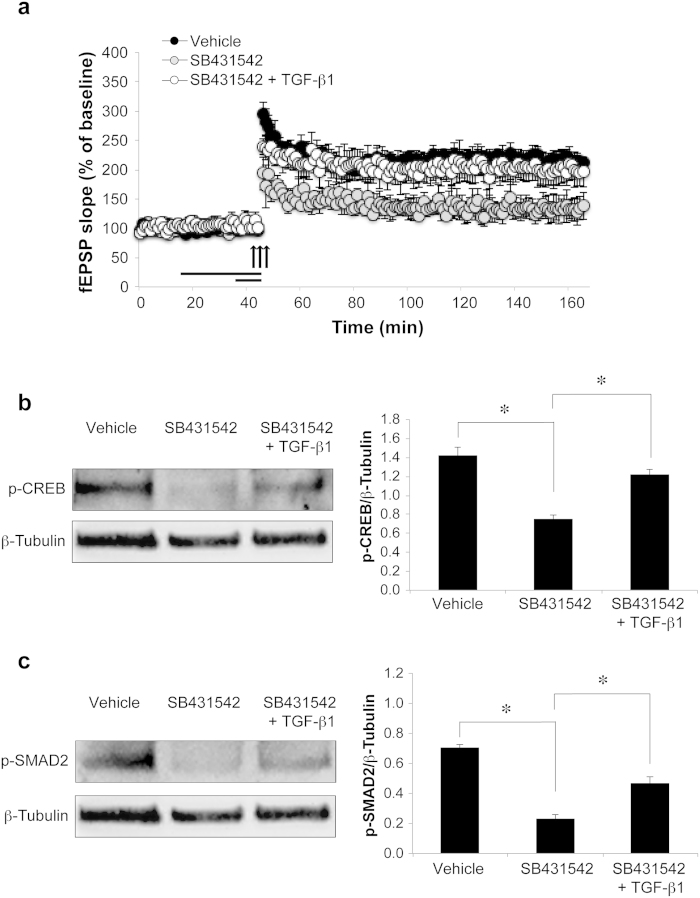
Inhibition of TGF-β1 signaling impairs LTP and CREB phosphorylation. (**a**) The impairment of LTP induced by perfusion with SB431542 (thirty minutes before tetanus) is rescued by TGF-β1 (ten minutes before tetanus). (**b**) Western blot analyses shows that SB431542 treatment blocks the physiological phosphorylation of CREB expected after a strong tetanus, whereas TGF-β1 rescues p-CREB levels in SB431542-treated tetanized slices. (**c**) Western blot analyses shows that SB431542 treatment induces a decrease of phospho-SMAD2 (ser465/467) whose levels are partially rescued by TGF-β1. *significant difference (p < 0.05).

To understand whether endogenous TGF-β1 plays a role in LTP, we blocked its signaling pathway by using SB431542 (20 μM)^15^, a selective inhibitor of activin-like kinase (ALK) 5 TGF-β type I receptor. The inhibition of L-LTP induced by SB431542 (20 μM) thirty min before a strong tetanus (n = 7/7; 138.45 ± 23.01 vs. 211.72 ± 7.46% of baseline slope 120 min after tetanus) was rescued when slices were concomitantly perfused with TGF-β1 (10 ng/ml) for 10 minutes before a strong tetanus (n = 7; 196.58 ± 25.69% of baseline slope 120 min after tetanus; ANOVA: F_(2, 18)_ = 10.495, p = 0.001 among the 3 groups; Fig. 2a) confirming that the impairment of LTP was specifically mediated by TGF-β1 inhibition.

SB431542 did not affect potentiation induced by a weak tetanus (n = 5/5; 125.68 ± 13.86 vs. 133.96 ± 4.23% of baseline slope 120 min after tetanus in vehicle-treated slices tetanized with a weak tetanus; ANOVA for repeated measures of the post-tetanic time points: F_(1, 8)_ = 0.046, p = 0.836 - data not shown).

Accordingly, western blot performed on hippocampal slices treated with SB431542 showed a decrease of the physiological phosphorylation of CREB expected after a strong tetanus (46% decrease of p-CREB in slices treated with SB431542 compared to vehicle; 0.74 ± 0.05 vs. 1.41 ± 0.09; one-way ANOVA: F_(2, 6)_ = 23.489, p = 0.001; Bonferroni post hoc: p = 0.002, Fig. 2b). TGF-β1 perfusion rescued p-CREB levels in SB431542-treated tetanized slices (1.21 ± 0.06; Bonferroni post hoc SB431542 + TGF-β1 vs. SB431542: p = 0.01; SB431542 + TGF-β1 vs. vehicle: p = 0.271; Fig. 2b).

We finally confirmed that SB431542 inhibited the phosphorylation of SMAD2 (Ser465/Ser467), an intracellular mediator of TGF-β1 signaling^8^,^17^. Indeed, SB431542 induced a 68% decrease of phospho-SMAD2 (p-SMAD2 Ser465/Ser467) levels compared to vehicle (0.70 ± 0.02 vs. 0.22 ± 0.05; one-way ANOVA: F_(2, 6)_ = 32.609, p = 0.001; Bonferroni post hoc: p = 0.001; Fig. 2c). p-SMAD2 levels were partially rescued by TGF-β1 treatment (0.46 ± 0.04; Bonferroni post hoc SB431542 + TGF-β1 vs. SB431542: p = 0.013; SB431542 + TGF-β1 vs. vehicle: p = 0.033; Fig. 2c).

### Selective inhibition of endogenous TGF-β1 signaling impairs object recognition memory

**Figure 3:**
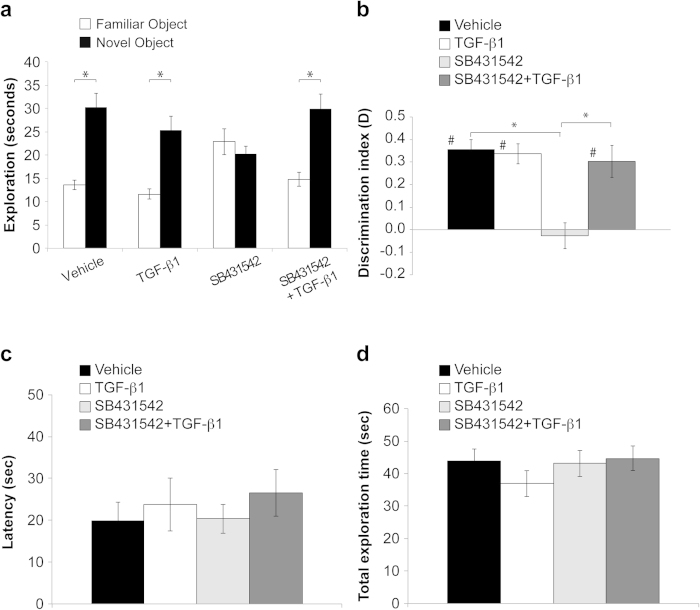
SB431542-induced impairment of object recognition memory is rescued by TGF-β1. (**a**) Exploration times of familiar and novel object during T2 (after a 24-h retention interval) show that mice treated with vehicle or TGF-β1 alone spend longer exploring the novel object, indicating an ability to learn. Animals treated with SB431542 display an impairment of memory (same amount of time exploring the familiar versus the novel object) that is rescued by concomitant treatment with TGF-β1 (greater amount of time in exploring the novel object in T2). (**b**) Analyses of the discrimination index (D) confirms that the SB431542-induced impairment of recognition memory is rescued by TGF-β1. A difference from 0 is depicted with hashes (#p < 0.05) (**c**) Latency to first approach to the novel object and (**d**) total exploration time is comparable in the 4 groups of mice.*significant difference (p < 0.05); #difference from 0.

We then studied recognition memory, a task based on the natural tendency of rodents to explore unfamiliar objects which, as recently documented by an increasing number of studies, depends on hippocampus integrity^18^,^19^. For this purpose we performed Object Recognition Test (ORT)^20^,^21^. The first day (T1) mice underwent a training in which they were presented with two identical objects; after 24 h (T2), the arena contained two objects: one was the same object as in T1 and the second was a new object. This 24 h inter-trial delay allowed us to measure long-term memory. The exploration time of the familiar and the novel object was recorded in T2. Mice were treated with i.p. injections of vehicle, TGF-β1, SB431542, or SB431542 + TGF-β1 30 min before T1. Mice treated with SB431542 (4.2 mg/kg) spent the same amount of time exploring the familiar compared with the novel object (n = 14; 22.91 ± 2.79 vs. 20.22 ± 1.74 seconds of exploration time familiar vs. novel object; paired t-test: t_(13)_ = 1.143, p = 0.274; Fig. 3a). However, a concomitant treatment with i.p. injection of TGF-β1 (1 μg) rescued object recognition memory as demonstrated by the greater amount of time in exploring the novel object in T2 (14.79 ± 1.46 vs. 29.89 ± 3.29 seconds of exploration time familiar vs. novel object; paired t-test: t_(12)_ = 4.193, p < 0.001; Fig. 3a). Vehicle- and TGF-β1-treated mice showed a good recognition memory (n = 13/13; Vehicle: 13.65 ± 1.04 vs. 30.26 ± 3.05 seconds of exploration; paired t-test: t_(12)_ = 5.982, p < 0.0001; TGF-β1: 11.65 ± 1.05 vs. 25.24 ± 3.06 seconds of exploration; paired t-test: t_(12)_ = 5.677, p < 0.0001; Fig. 3a).

These findings were confirmed by the analyses of the discrimination index, D (one-way ANOVA: F_(3, 49)_ = 10.499, p < 0.0001; two-way ANOVA for the interaction between SB431542 and TGF-β1: F_(2, 37)_ = 11.515, p < 0.0001; Fig. 3b). SB431542-treated animals showed an impairment of learning since their D did not significantly differ from zero (t_(13)_ = 0.481, p = 0.639), as opposed to the one of vehicle (t_(12)_ = 7.866, p < 0.0001) or TGF-β1-treated mice (t_(12)_ = 7.33, p < 0.0001). SB431542-induced impairment of memory was selectively rescued by TGF-β1 treatment (D vs. zero: t_(12)_ = 4.104, p = 0.001; Bonferroni post hoc SB431542 vs. SB431542 + TGF-β1: p = 0.033; Fig. 3b). The 4 groups of mice did not show differences in the latency to first approach to the novel object (one-way ANOVA: F_(3, 49)_ = 0.363, p = 0.780; two-way ANOVA for SB431542 and TGF-β1 interaction: F_(2, 37)_ = 0.342, p = 0.713; Fig. 3c) nor in total exploration time (one-way ANOVA: F_(3, 49)_ = 0.795, p = 0.502; two-way ANOVA for for SB431542 and TGF-β1 interaction: F_(2, 37)_ = 1.041, p = 0.363; Fig. 3d).

**Figure 4:**
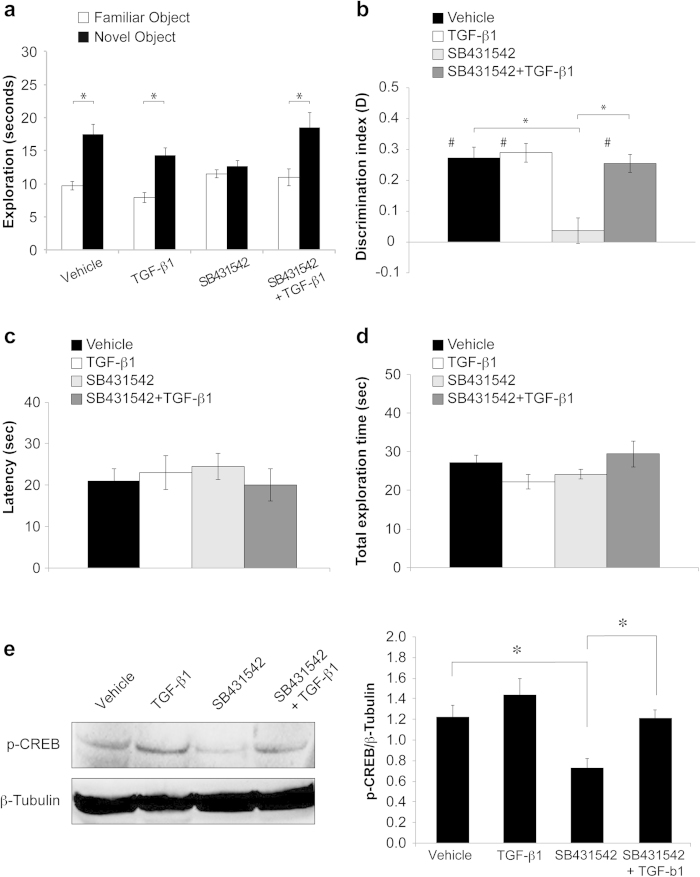
Intrahippocampal injections of TGF-β1 rescue the SB431542-induced impairment of object recognition memory and CREB phosphorylation. (**a**) Intrahippocampal injections of SB431542 induce an impairment of recognition memory (same amount of exploration times for the familiar and the novel object) that is rescued by concomitant treatment with TGF-β1. A significant difference in exploration times is also present in animals treated with vehicle or TGF-β1 alone. (**b**) The decrease of discrimination index (D) in SB431542-injected mice (compared to vehicle) is rescued by TGF-β1. D is significantly different from zero (#) in mice treated with vehicle, TGF-β1 or SB431542 + TGF-β1, indicating their ability to learn. (**c**) Latency to first approach to the novel object and (**d**) total exploration time is comparable in the 4 groups of mice. (**e**) Western blot analyses shows that the SB431542-induced decrease of p-CREB levels is restored by TGF-β1 treatment. *significant difference (p < 0.05); #difference from 0.

We then investigated whether direct injections of TGF-β1 or SB431542 into the hippocampus might affect memory. Cannulas were implanted bilaterally into the mouse dorsal hippocampi and, after 6 – 8 days of recovery from surgery, animals underwent ORT using the same protocol described above. Vehicle, TGF-β1 (3.3 ng over 1 μl), SB431542 (38.4 ng over 1 μl) or SB431542 + TGF-β1 were injected right before T1. Both vehicle- and TGF-β1-treated mice took longer exploring the novel object in T2 (n = 8/8; Vehicle: 9.70 ± 0.69 vs. 17.40 ± 1.58 seconds of exploration time familiar vs. novel object; paired t-test: t_(7)_ = 4.523, p = 0.003; TGF-β1: 7.94 ± 0.77 vs. 14.27 ± 1.17 seconds of exploration time familiar vs. novel object; paired t-test: t_(7)_ = 2.632, p < 0.0001; Fig. 4a). Mice treated with SB431542 spent the same amount of time exploring the familiar versus the novel object, thus indicating an impairment of recognition memory (n = 8; 11.51 ± 0.60 vs. 12.62 ± 0.95% seconds of exploration; paired t-test: t_(7)_ = 0.831, p = 0.434; Fig. 4a). Concomitant administration of exogenous TGF-β1 rescued the SB431542-induced impairment as indicated by a longer time spent in exploring the novel object (n = 8; 10.97 ± 1.28 vs. 18.45 ± 2.26 seconds of exploration; paired t-test: t_(7)_ = 3.808, p = 0.007; Fig. 4a). As shown in Fig. 4b, the discrimination index (D) confirmed that mice treated with vehicle, TGF-β1 or SB431542 + TGF-β1 recognized the familiar object, because D significantly differed from 0 in each group (vehicle: t_(7)_ = 6.077, p = 0.001; TGF-β1: t_(7)_ = 7.182, p < 0.0001; TGF-β1 + SB431542: t_(7)_ = 6.624, p < 0.0001), as opposed to SB431542 (t_(7)_ = 0.664, p = 0.528). A comparison of the 4 groups by one-way ANOVA confirmed that the SB431542-induced impairment of recognition memory was rescued by TGF-β1 treatment (F_(3, 28)_ = 6.958, p = 0.001; Bonferroni post hoc SB431542 vs. SB431542 + TGF-β1: p = 0.011; Fig. 4b). Two-way ANOVA showed an interaction between TGF-β1 and SB431542 treatment (F_(2, 21)_ = 9.253, p = 0.001). No differences were detected in latency to first approach to the novel object (one-way ANOVA: F_(3, 28)_ = 1.12, p = 0.358; two-way ANOVA for SB431542 and TGF-β1 interaction: F_(2, 21)_ = 0.206, p = 0.815; Fig. 4c) and total exploration time (one-way ANOVA: F_(3, 49)_ = 1.034, p = 0.393; two-way ANOVA for SB431542 and TGF-β1 interaction: F_(2, 21)_ = 1.462, p = 0.254; Fig. 4d).

We then wanted to verify whether the changes of recognition memory obtained after intrahippocampal injections *in vivo* correlated with p-CREB levels. After behavioral studies, mice hippocampi were dissected and used for western blot analyses. One-way ANOVA showed a difference among the 4 groups (F_(3, 16)_ = 7.252, p = 0.003; Fig. 4e). In particular, SB431542 treatment induced a 40% decrease of p-CREB compared to vehicle (0.73 ± 0.08 vs 1.22 ± 0.10; Bonferroni post hoc p < 0.0001; Fig. 4e) that was rescued by TGF-β1 (1.21 ± 0.08; Bonferroni post hoc p = 0.013; Fig. 4e). Two-way ANOVA showed an interaction between SB431542 and TGF-β1 treatment (F_(2, 12)_ = 10.425, p = 0.002).

## Discussion

TGF-β1 is an anti-inflammatory cytokine, which has been demonstrated to have neurotrophic and neuroprotective properties^8^,^17^. Here we provide novel evidence that TGF-β1 has a role in physiological mechanisms underlying synaptic plasticity and memory in the hippocampus.

We first showed that treatment of hippocampal slices with TGF-β1 converted E-LTP into L-LTP, consistent with previous reports indicating that TGF-β1 induced long-term but not short-term facilitation in Aplysia ganglia^11^, where it also increased excitability and decreased the firing threshold of cultured sensory neurons^12^.

In our electrophysiological experiments, the effective dose of TGF-β1 was 10 ng/ml, as in previous works indicating that 10 ng/ml of TGF-β1 was able to significantly increase p-CREB in sensory neurons of Aplysia^7^, whereas we did not observe any effect with 1 ng/ml or 5 ng/ml. We also demonstrated that endogenous TGF-β1 plays a role in LTP. Indeed, inhibition of TGF-β1 signaling by SB431542, a selective inhibitor of ALK5 TGF-β type I receptor, impaired L-LTP induced by a strong tetanization, whereas it did not modify potentiation after a weak tetanus. Rescue experiments by the addition of TGF-β1 concomitant to SB431542 restored LTP, confirming the role of endogenous TGF-β1 signaling in LTP.

These electrophysiological findings prompted us to investigate the role of TGF-β1 in long-term memory by ORT, a test relying on the function of the hippocampus^18^,^19^, the perirhinal and the medial temporal lobe cortices, widely used to evaluate the neural basis of memory^20^,^21^. As for LTP, the inhibition of TGF-β1 signaling obtained after i.p. as well as intrahippocampal administration of SB431542 caused a severe impairment of recognition memory that was completely rescued by TGF-β1, providing a novel role for TGF-β1 in learning and memory. Few other studies have investigated the involvement of TGF-β1 on hippocampal memory demonstrating that it was able to rescue cognitive impairment in pilocarpine-treated rats^22^ and that TGF-β1 hippocampal overexpression restored neurogenesis as well as recognition memory in models of LPS-induced inflammation^23^. Other members of the TGF-β superfamily have a similar role. Indeed, TGF-β2 facilitated synaptic plasticity in rat hippocampal neurons^14^, while Activin has been shown to be required for hippocampal L-LTP, consolidation of long-term memory^24^, and induction of moderate LTP upon weak theta-burst stimulation by acting on NMDA receptors currents and spine density^25^.

Another relevant finding of this study is the modification of CREB phosphorylation, which has been shown to play a central role in LTP and memory^4^,^5^,^6^. Our *in vitro* and *in vivo* studies showed that the inhibition of TGF-β1 signaling induced by SB431542 provoked a decrease of hippocampal p-CREB levels that was rescued by concomitant treatment with TGF-β1. Moreover, TGF-β1 treatment stimulated CREB phosphorylation in hippocampal slices after a weak tetanus. These findings correlated with the modifications of synaptic plasticity and memory and are consistent with previous works demonstrating that TGF-β1 increased nuclear levels of p-CREB in Aplysia^7^, whereas TGF-β2-induced long-term changes in excitatory and miniature post-synaptic currents in the hippocampus^14^.

In this work we also investigate the involvement of p-SMAD2, a TGF-β1 downstream pathway effector. Indeed, members of the TGF superfamily act through a receptor complex constituted by the serine/threonine receptors ALK/TGF-β type I receptor and TGF-β type II receptor (TβRII), strongly expressed in the CNS and in particular in the hippocampus^26^. TGF-β1 binding to TβRII induces the assembly of type I and type II receptors into a complex, with the subsequent transphosphorylation of type I receptor by the type II receptor kinase. The consequent activation of type I receptor leads to phosphorylation of selected SMAD proteins that, in turn, translocate into the nucleus to regulate the expression of different target genes involved in cell proliferation and neuronal survival^6^. Here we confirmed that SB431542, a validated tool to evaluate cellular mechanisms mediated by endogenous TGF-β1^15^, selectively induced an inhibition of p-SMAD2 signaling, which was rescued by TGF-β1 treatment, suggesting a similar trend between p-SMAD2 and p-CREB levels. This connection between the two pathways could be relevant considering the role of TGF-β1 in neurodegenerative disorders characterized by synaptic plasticity and memory loss, such as Alzheimer’s Disease (AD). Indeed, an impairment of TGF-β1 has been demonstrated in the AD brain and serum^17^ and a nucleotide polymorphism of the TGF-β1 gene has been associated with an increased conversion of mild cognitive impairment in AD and with an increased risk to develop Late-Onset AD^27^. Accordingly, TGF-β1 signaling has been found to interact at different levels with Amyloid-beta peptide (Aβ), a key factor in the pathogenesis of AD. TGF-β1 protected against Aβ-induced neurodegeneration^16^ and, on the other hand, a suppression of TGF-β1 pathway amplified Aβ neurotoxicity in rat hippocampus^16^,^17^. Thus, the neuroprotective features of TGF-β1 combined with its physiological activity on hippocampal synaptic plasticity and memory suggest that it might represent a new therapeutical strategy against neurodegenerative diseases characterized by an impairment of TGF-β signaling, such as AD.

## Materials and Methods

### Animals

All experiments have been performed on 5-months old C57BL/6 mice obtained from a breeding colony kept at the University of Catania. Mice were maintained with a controlled temperature (21 °C ± 1 °C) and humidity (50%) on a 12 h light/dark cycle, with ad libitum food and water. All animal experimentation was conducted in accordance with the guidelines laid down by the European Community Council (2010/63/EU). The experimental protocols have been approved by the University Institutional Animal Care and Use Committee (Project #204/2013, Palmeri/Puzzo).

### Drugs

SB431542^13^ (Tocris, Bristol, UK) was reconstituted in DMSO (20 mM), dissolved in PBS containing 2% DMSO and applied to hippocampal slices (20 μM in ACSF) or administered via intraperitoneal injection at a concentration of 4.2 mg/kg according to previous studies^28^. Human TGF-β1 (R&D system, Minneapolis, MN, USA) was reconstituted at 20 μg/mL in sterile 4 mM HCl containing 1 mg/mL bovine serum albumin, dissolved in ACSF for *in vitro* studies (1, 5 or 10 ng/ml) and in PBS for *in vivo* studies (1 μg in 300 μl PBS)^7^,^16^. We injected 38.4 ng of SB431542 and 3.3 ng of TGF-β1 over 1 μl for intrahippocampal treatment.

### Electrophysiology

Electrophysiological recordings were performed on 400 μm transverse slices, in CA1 hippocampal area, as previously described^29^,^30^. Slices were maintained in a recording chamber at 29 °C and perfused with artificial cerebrospinal fluid (ACSF composition in mM: 124.0 NaCl, 4.4 KCl, 1.0 Na2HPO4, 25.0 NaHCO3, 2.0 CaCl2, 2.0 MgCl2, 10.0 glucose) continuously bubbled with 95% O2 and 5% CO2. Field excitatory postsynaptic potentials (fEPSPs) were recorded by stimulating Schaffer collateral fibers with a bipolar tungsten electrode and recording in CA1 stratum radiatum with a glass electrode filled with ACSF. After evaluation of basal synaptic transmission, a 15 min baseline was recorded every minute at an intensity evoking approximately 35% of the maximum response. LTP was induced by 4 pulses at 100 Hz, with the bursts repeated at 5 Hz and 1 (weak tetanus) or 3 tetani (strong tetanus) of 10-burst trains. Responses were recorded for 2 hrs after tetanization and measured as fEPSP slope, expressed as percentage of baseline.

### Infusion technique

Cannulas were implanted as previously described^31^. After anesthesia with Tiletamine + Zolazepam (60 mg/kg) and Medetomidine (40 μg/kg), mice were implanted with a 26-gauge guide cannula into the dorsal part of the hippocampi (coordinates: posterior = 2.46 mm, lateral = 1.50 mm to a depth of 1.30 mm). The cannulas were fixed to the skull with acrylic dental cement (RelyX™ Unicem). After 6–8 days of recovery, drugs were injected bilaterally in a final volume of 1 μl over 1 min through infusion cannulas that were connected to a microsyringe by a polyethylene tube. Mice were injected right before the training session (T1) of the ORT. During infusion, animals were handled gently to minimize stress. After infusion, the needle was left in place for another minute to allow diffusion. After behavioral testing, animals were killed and their hippocampi were removed, frozen, and then used for western blot analysis. In some animals a solution of 4% methylene blue was infused for histological localization of infusion cannulas.

### Novel object recognition test

Novel object recognition test was performed and analyzed as previously described^21^,^30^. After three days of habituation, at day 4th mice underwent the training session (T1). After treatment with vehicle, TGF-β1, SB431542 or TGF-β1 + SB431542 (via i.p. thirty min before T1, or via intrahippocampal injections right before T1) mice were placed in the arena for 10 min and allowed to explore two identical objects. Twenty-four hours after T1 mice underwent the second trial (T2) to test memory retention. Mice were presented with two different objects, respectively a “familiar” (i.e. the one used for T1) and a “novel” object. Animal exploration - defined as the mouse pointing its nose toward the object from a distance not >2 cm – was evaluated in T2 to analyze: (i) exploration of the novel and of the familiar object; (ii) discrimination (D) index calculated as “exploration of novel object minus exploration of familiar object/total exploration time”; (iii) latency to first approach to novel object; (iv) total exploration time.

### Western blot

Western blot analyses was performed as previously described^30^ on hippocampal slices previously treated as for electrophysiological experiment and stored in liquid nitrogen at 1 min after treatment or in hippocampi of animals previously treated with intrahippocampal injections. Tissues were then homogenized in RIPA buffer in the presence of a cocktail of protease inhibitors (Sigma P2714), serine/threonine phosphatase inhibitors (Sigma P0044) and tyrosine protein phosphatases inhibitors (Sigma P5726) and sonicated. Protein concentrations were determined by Bradford’s method using bovine serum albumin as a standard. After blocking, membranes were incubated with primary antibodies overnight at 4 °C: rabbit anti-phospho-CREB (Ser133) (Millipore, Billerica, MA, USA; 1:1000), rabbit anti-phospho-SMAD2 (ser465/467) (Cell signaling Technology, Danvers, MA; 1:1000) and mouse anti-β-tubulin (Sigma-Aldrich; 1:500). Proteins were visualized using the enhancing chemiluminescence detection system SuperSignal (Pierce, USA). Chemiluminescence was detected by the Uvitec System (Cambridge, UK) and quantified by densitometric analysis in three different blots per experiment.

### Statistical Analysis

All experiments were blind with respect to treatment. Data were expressed as mean ± standard error mean (SEM). Statistical analyses was performed by using Systat software (Chicago, IL, USA). For LTP we used ANOVA with repeated measures comparing 120 minutes of recording after tetanus; in some experiments we used one-way ANOVA with Bonferroni post-hoc to compare the 120° min of recording among groups. For ORT we used one-way ANOVA with Bonferroni post-hoc for comparisons among groups, paired t-test to compare exploration of the novel vs. the familiar object in the same mouse, one sample t-test to compare D with zero. For western blot we used one-way ANOVA with Bonferroni post-hoc and two-way ANOVA for tetanus and treatment. The level of significance was set at p < 0.05.

## Additional Information

**How to cite this article**: Caraci, F. *et al.* A key role for TGF-β1 in hippocampal synaptic plasticity and memory. *Sci. Rep.* **5**, 11252; doi: 10.1038/srep11252 (2015).

## References

[b1] Bliss T.V. & Collingridge G.L. A synaptic model of memory: long-term potentiation in the hippocampus. Nature. 361, 31–9 (1993). 8421494 10.1038/361031a0

[b2] Izquierdo I. *et al.* Different molecular cascades in different sites of the brain control memory consolidation. Trends Neurosci. 29, 496–505 (2006). 16872686 10.1016/j.tins.2006.07.005

[b3] Bollen E., *et al.* Improved Long-Term Memory via Enhancing cGMP-PKG Signaling Requires cAMP-PKA Signaling. Neuropsychopharmacology. 39, 2497–505 (2014). 24813825 10.1038/npp.2014.106 PMC4207334

[b4] Josselyn S.A. & Nguyen P.V. CREB, synapses and memory disorders: past progress and future challenges. Curr. Drug Targets CNS Neurol. Disord. 4, 481–97 (2005). 10.2174/156800705774322058 16266283

[b5] Kida S. A Functional Role for CREB as a Positive Regulator of Memory Formation and LTP. Exp. Neurobiol. 21, 136–40 (2012). 23319873 10.5607/en.2012.21.4.136 PMC3538177

[b6] Teich A.F. *et al.* Synaptic Therapy in Alzheimer’s Disease: A CREB-centric Approach. Neurotherapeutics 12, 29–41 (2015). 25575647 10.1007/s13311-014-0327-5 PMC4322064

[b7] Chin J., Liu R.Y., Cleary L.J., Eskin A. & Byrne J.H. TGF-beta1-induced long-term changes in neuronal excitability in aplysia sensory neurons depend on MAPK. J. Neurophysiol. 95, 3286–90 (2006). 16617179 10.1152/jn.00770.2005

[b8] ten Dijke P. & Hill C.S. New insights into TGF-beta-Smad signalling. Trends Biochem. Sci. 29, 265–73 (2004). 15130563 10.1016/j.tibs.2004.03.008

[b9] Specht H. *et al.* Transforming growth factor beta2 is released from PC12 cells via the regulated pathway of secretion. Mol. Cell Neurosci. 22, 75–86 (2003). 12595240 10.1016/s1044-7431(02)00023-4

[b10] Lacmann A., Hess D., Gohla G., Roussa E. & Krieglstein K. Activity-dependent release of transforming growth factor-beta in a neuronal network *in vitro*. Neuroscience. 150, 647–57 (2007). 17997227 10.1016/j.neuroscience.2007.09.046

[b11] Zhang F., Endo S., Cleary L.J., Eskin A. & Byrne J.H. Role of transforming growth factor-beta in long-term synaptic facilitation in Aplysia. Science. 275, 1318–20 (1997). 9036859 10.1126/science.275.5304.1318

[b12] Chin J., Angers A., Cleary L.J., Eskin A. & Byrne J.H. TGF-beta1 in Aplysia: role in long-term changes in the excitability of sensory neurons and distribution of TbetaR-II-like immunoreactivity. Learn. Mem. 6, 317–30 (1999). 10492013 PMC311291

[b13] Chin J., Angers A., Cleary L.J., Eskin A. & Byrne JH. Transforming growth factor beta1 alters synapsin distribution and modulates synaptic depression in Aplysia. J. Neurosci. 22, RC220 (2002). 11978861 10.1523/JNEUROSCI.22-09-j0004.2002 PMC6758346

[b14] Fukushima T., Liu R.Y. & Byrne JH. Transforming growth factor-beta2 modulates synaptic efficacy and plasticity and induces phosphorylation of CREB in hippocampal neurons. Hippocampus. 17, 5–9 (2007). 17094084 10.1002/hipo.20243

[b15] Laping N.J. *et al.* Inhibition of transforming growth factor (TGF)-beta1-induced extracellular matrix with a novel inhibitor of the TGF-beta type I receptor kinase activity: SB-431542. Mol. Pharmacol. 62, 58–64 (2002). 12065755 10.1124/mol.62.1.58

[b16] Caraci F. *et al.* TGF-beta 1 protects against Abeta-neurotoxicity via the phosphatidylinositol-3-kinase pathway. Neurobiol. Dis. 30, 234–42 (2008). 18356065 10.1016/j.nbd.2008.01.007

[b17] Caraci F. *et al.* TGF-β1 pathway as a new target for neuroprotection in Alzheimer’s disease. CNS Neurosci. Ther. 17, 237–49 (2011). 19925479 10.1111/j.1755-5949.2009.00115.x PMC6493850

[b18] Broadbent N.J., Gaskin S., Squire L.R. & Clark R.E. Object recognition memory and the rodent hippocampus. Learn. Mem. 17, 5–11 (2009). 20028732 10.1101/lm.1650110 PMC2807177

[b19] Barker G.R. & Warburton E.C. When is the hippocampus involved in recognition memory? J. Neurosci. 31, 10721–10731 (2011). 21775615 10.1523/JNEUROSCI.6413-10.2011 PMC6622630

[b20] Antunes M. & Biala G. The novel object recognition memory: neurobiology, test procedure, and its modifications. Cogn. Process. 13, 93–110 (2012). 22160349 10.1007/s10339-011-0430-z PMC3332351

[b21] van Goethem N.P. *et al.* Object recognition testing: rodent species, strains, housing conditions, and estrous cycle. Behav. Brain Res. 232, 323–34 (2012). 22481082 10.1016/j.bbr.2012.03.023

[b22] Li L.Y. *et al.* TGFβ1 treatment reduces hippocampal damage, spontaneous recurrent seizures, and learning memory deficits in pilocarpine-treated rats. J. Mol. Neurosci. 50, 109–23 (2013). 22936246 10.1007/s12031-012-9879-1

[b23] Graciarena M., Depino A.M. & Pitossi F.J. Prenatal inflammation impairs adult neurogenesis and memory related behavior through persistent hippocampal TGFβ1 downregulation. Brain Behav. Immun. 24, 1301–9 (2010). 20600816 10.1016/j.bbi.2010.06.005

[b24] Ageta H. *et al.* Activin plays a key role in the maintenance of long-term memory and late-LTP. Learn. Mem. 17, 176–85 (2010). 20332189 10.1101/lm.16659010

[b25] Hasegawa Y. *et al.* Acute modulation of synaptic plasticity of pyramidal neurons by activin in adult hippocampus. Front. Neural Circuits. 8, 56 (2014). 24917791 10.3389/fncir.2014.00056 PMC4040441

[b26] Vivien D. & Ali C. Transforming growth factor-beta signalling in brain disorders. Cytokine Growth Factor Rev. 17, 121–8 (2006). 16271500 10.1016/j.cytogfr.2005.09.011

[b27] Caraci F. *et al.* The CC genotype of transforming growth factor-β1 increases the risk of late-onset Alzheimer’s disease and is associated with AD-related depression. Eur. Neuropsychopharmacol. 22, 281–9 (2012). 21924590 10.1016/j.euroneuro.2011.08.006

[b28] Ma B. *et al.* Inhibition of activin receptor-like kinase 5 induces matrix metallopeptidase 9 expression and aggravates lipopolysaccharide-induced pulmonary injury in mice. Eur. Rev. Med. Pharmacol. Sci. 17, 1051–9 (2013). 23661518

[b29] Ricciarelli R. *et al.* A novel mechanism for cyclic adenosine monophosphate-mediated memory formation: Role of amyloid beta. Ann. Neurol. 75, 602–7 (2014). 24591104 10.1002/ana.24130

[b30] Puzzo D. *et al.* F3/Contactin promotes hippocampal neurogenesis, synaptic plasticity, and memory in adult mice. Hippocampus. 23, 1367–82 (2013). 23939883 10.1002/hipo.22186

[b31] Puzzo D., Privitera L. & Palmeri A. Hormetic effect of amyloid-β peptide in synaptic plasticity and memory. Neurobiol. Aging 33, 1484.e15-24 (2012). 10.1016/j.neurobiolaging.2011.12.020 22284988

